# Supplementary material for: Effects of social organization, trap arrangement and density, sampling scale, and population density on bias in population size estimation using some common mark-recapture estimators
Source: PLoS One. 2017 Mar 17;12(3):e0173609. doi: 10.1371/journal.pone.0173609 (PMC5357017; doi:10.1371/journal.pone.0173609)
Supplement: S1 Appendix — This appendix contains ANOVA tables for all four- and five-way ANOVAs, as also tables listing the mean relative bias for various combinations of factor-levels, along with the pattern of significance among them in pair-wise multiple comparisons. Mean values and significance levels in pair-wise comparisons are shown only for main effects and interactions that had a significant effect in the ANOVA. (DOC) [file pone.0173609.s001.doc]

**Supporting Material**

**Appendix S1**

**Results of analyses of the effects of trap arrangement, trap density, sampling scale, social organization and adult density on relative bias in population size estimation using three mark-recapture estimators. This appendix contains ANOVA tables for all four- and five-way ANOVAs, as also tables listing the mean relative bias for various combinations of factor-levels, along with the pattern of significance among them in pair-wise multiple comparisons. Mean values of relative bias and significance levels in pair-wise comparisons are shown only for main effects and interactions that had a significant effect in the ANOVA. Also contained in this appendix are figures, and a table with significance and effect size patterns for the analysis with initial adult density as a factor.**

**TABLE A: Results of post hoc comparisons of fixed factor levels from the POPAN analysis using actual adult density, showing relative bias means and patterns of significance. Post hoc comparisons were naturally carried out only for those factors that were significant in the four-way ANOVA. TA: trap arrangement.**

**TABLE B: Results of post hoc comparisons of interactions from the POPAN analysis using actual adult density, showing relative bias means and patterns of significance. Post hoc comparisons were naturally carried out only for those interactions that were significant in the four-way ANOVA. TA: trap arrangement.**

**TABLE C:**

**Results of post hoc comparisons of fixed factor levels from the POPAN analysis using initial adult density, showing relative bias means and patterns of significance. Post hoc comparisons were naturally carried out only for those factors that were significant in the four-way ANOVA. TA: trap arrangement.**

**TABLE D: Results of post hoc comparisons of interactions from the POPAN analysis using initial adult density, showing relative bias means and patterns of significance. Post hoc comparisons were naturally carried out only for those interactions that were significant in the four-way ANOVA. TA: trap arrangement**

**TABLE E: Results of post hoc comparisons of fixed factor levels from the Robust Design analysis using actual adult density, showing relative bias means and patterns of significance. Post hoc comparisons were naturally carried out only for those factors that were significant in the four-way ANOVA. TA: trap arrangement.**

**TABLE F: Results of post hoc comparisons of interactions from the Robust Design analysis using actual adult density, showing relative bias means and patterns of significance. Post hoc comparisons were naturally carried out only for those interactions that were significant in the four-way ANOVA. TA: trap arrangement.**

**TABLE G: Results of post hoc comparisons of fixed factor levels from the Robust Design analysis using initial adult density, showing relative bias means and patterns of significance. Post hoc comparisons were naturally carried out only for those factors that were significant in the four-way ANOVA. TA: trap arrangement.**

**TABLE H:**

**Results of post hoc comparisons of interactions from the Robust Design analysis using initial adult density, showing relative bias means and patterns of significance. Post hoc comparisons were naturally carried out only for those interactions that were significant in the four-way ANOVA. TA: trap arrangement.**

**TABLE I: Results of post hoc comparisons of fixed factor levels from the Robust Design with Heterogeneity analysis using actual adult density, showing relative bias means and patterns of significance. Post hoc comparisons were naturally carried out only for those factors that were significant in the four-way ANOVA. TA: trap arrangement.**

**TABLE J: Results of post hoc comparisons of interactions from the Robust Design with Heterogeneity analysis using actual adult density, showing relative bias means and patterns of significance. Post hoc comparisons were naturally carried out only for those interactions that were significant in the four-way ANOVA. TA: trap arrangement.**

**TABLE K: Results of post hoc comparisons of fixed factor levels from the Robust Design with Heterogeneity analysis using initial adult density, showing relative bias means and patterns of significance. Post hoc comparisons were naturally carried out only for those factors that were significant in the four-way ANOVA. TA: trap arrangement.**

**TABLE L: Results of post hoc comparisons of interactions from the Robust Design with Heterogeneity analysis using initial adult density, showing relative bias means and patterns of significance. Post hoc comparisons were naturally carried out only for those interactions that were significant in the four-way ANOVA. TA: trap arrangement.**

**TABLE M: Five-way ANOVA table for the POPAN analysis with actual adult density as a factor.**

**TABLE N: Five-way ANOVA table for the POPAN analysis with initial adult density as a factor.**

**TABLE O: Five-way ANOVA table for the Robust Design analysis with actual adult density as a factor.**

**TABLE P: Five-way ANOVA table for the Robust Design analysis with initial adult density as a factor.**

**TABLE Q: Five-way ANOVA table for the Robust Design with Heterogeneity analysis with actual adult density as a factor.**

**TABLE R: Five-way ANOVA table for the Robust Design with Heterogeneity analysis with initial adult density as a factor.**

**TABLE S: Four-way ANOVA table for the POPAN analysis within the uniform trap arrangement case with actual adult density as a factor.**

**TABLE T: Four-way ANOVA table for the POPAN analysis within the random trap arrangement case with actual adult density as a factor.**

**TABLE U: Four-way ANOVA table for the POPAN analysis within the uniform trap arrangement case with initial adult density as a factor.**

**TABLE V: Four-way ANOVA table for the POPAN analysis within the random trap arrangement case with initial adult density as a factor.**

**TABLE W: Four-way ANOVA table for the Robust Design analysis within the uniform trap arrangement case with actual adult density as a factor.**

**TABLE X: Four-way ANOVA table for the Robust Design analysis within the random trap arrangement case with actual adult density as a factor.**

**TABLE Y: Four-way ANOVA table for the Robust Design analysis within the uniform trap arrangement case with initial adult density as a factor.**

**TABLE Z: Four-way ANOVA table for the Robust Design analysis within the random trap arrangement case with initial adult density as a factor.**

**TABLE AA: Four-way ANOVA table for the Robust Design with Heterogeneity analysis within the uniform trap arrangement case with actual adult density as a factor.**

**TABLE AB: Four-way ANOVA table for the Robust Design with Heterogeneity analysis within the random trap arrangement case with actual adult density as a factor.**

**TABLE AC: Four-way ANOVA table for the Robust Design with Heterogeneity analysis within the uniform trap arrangement case with initial adult density as a factor.**

**TABLE AD: Four-way ANOVA table for the Robust Design with Heterogeneity analysis within the random trap arrangement case with initial adult density as a factor.**

**TABLE AE: The pattern of significance in the four-way fully-factorial ANOVAs done on relative bias in population size estimates using the three mark-recapture models using initial density as a factor (***=*P*<0.001, **=*P*<0.01, *=*P*<0.05, Blank cell=Not significant). Effect sizes (*η*^2^ = SS_Factor or Interaction_/SS_Total_) are shown as colour shading. TA:trap arrangement.**

|  | **POPAN** | | **Robust Design** | | **Robust Design with Heterogeneity** | |
| --- | --- | --- | --- | --- | --- | --- |
|  | Uniform TA | Random TA | Uniform TA | Random TA | Uniform TA | Random TA |
| **Trap Density (1)** | *** | *** | *** | *** | *** | *** |
| **Spatial Scale (2)** | *** |  | *** | *** | *** |  |
| **Social organi-zation (3)** | *** | *** | *** | *** | *** | *** |
| **Adult Density (4)** |  |  | *** | *** | *** | *** |
| **1x2** |  | *** | *** | *** | *** | *** |
| **1x3** | ** | *** | *** | *** | *** | *** |
| **2x3** | *** | *** | ** | *** | ** | *** |
| **1x4** |  |  | ** | *** | ** |  |
| **2x4** |  |  | *** | ** | *** | * |
| **3x4** | *** | *** | *** | *** | ** | ** |
| **1x2x3** |  |  | * | *** | *** | *** |
| **1x2x4** |  |  | * | *** | * |  |
| **1x3x4** |  |  |  | * |  |  |
| **2x3x4** |  |  | * | * |  | * |
| **1x2x3x4** |  | ** |  |  |  |  |


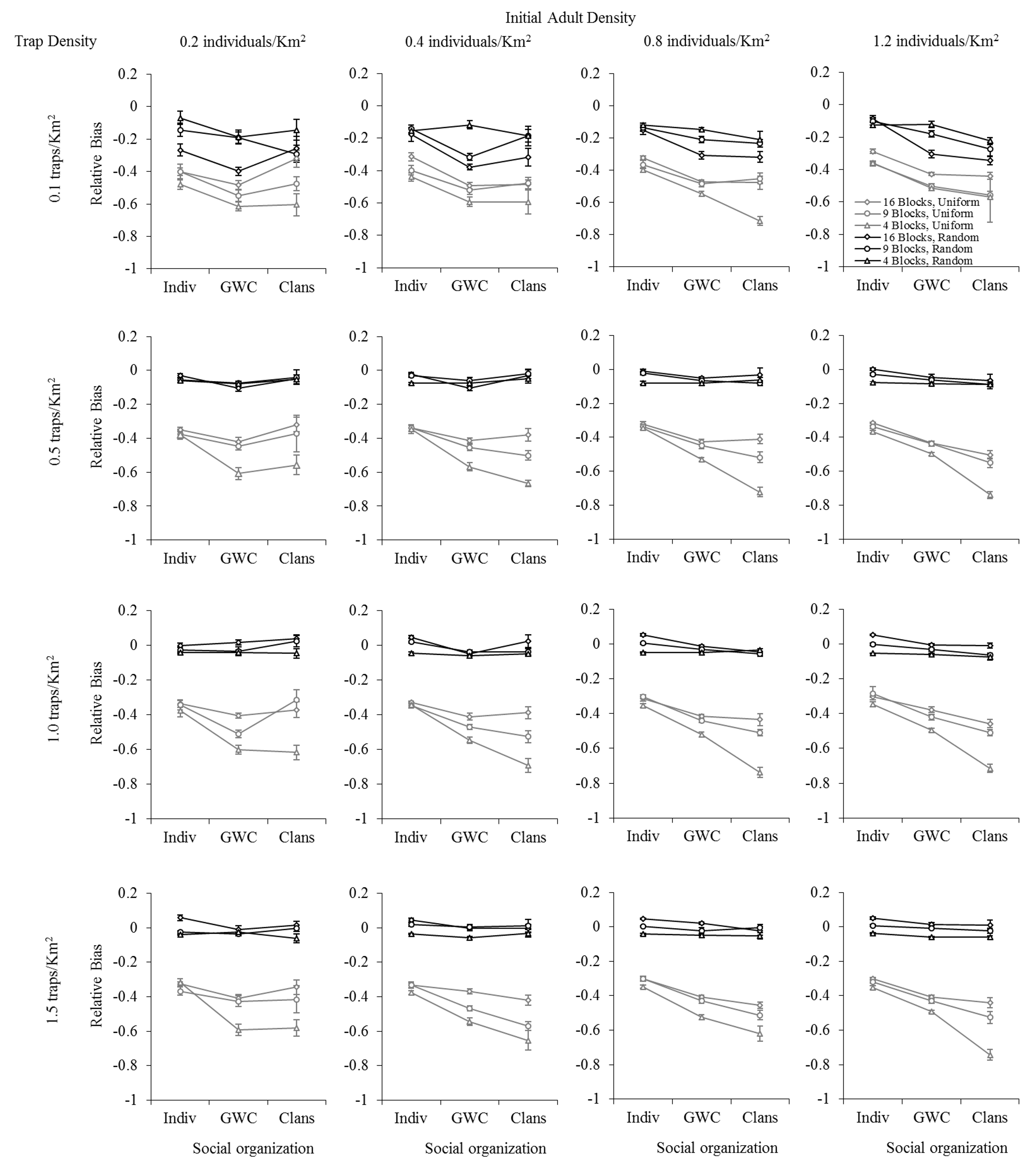
**FIGURE A: The effect of trap density, sampling scale, social organization, and adult density on mean relative bias in population size estimation using POPAN. Data shown are for uniform (grey lines) and random (black lines) trap arrangements, using initial adult densities. Error bars represent standard errors. Social organization: Indiv: non-associating individuals; GWC: groups within clans; Clans: fixed clans.**

**
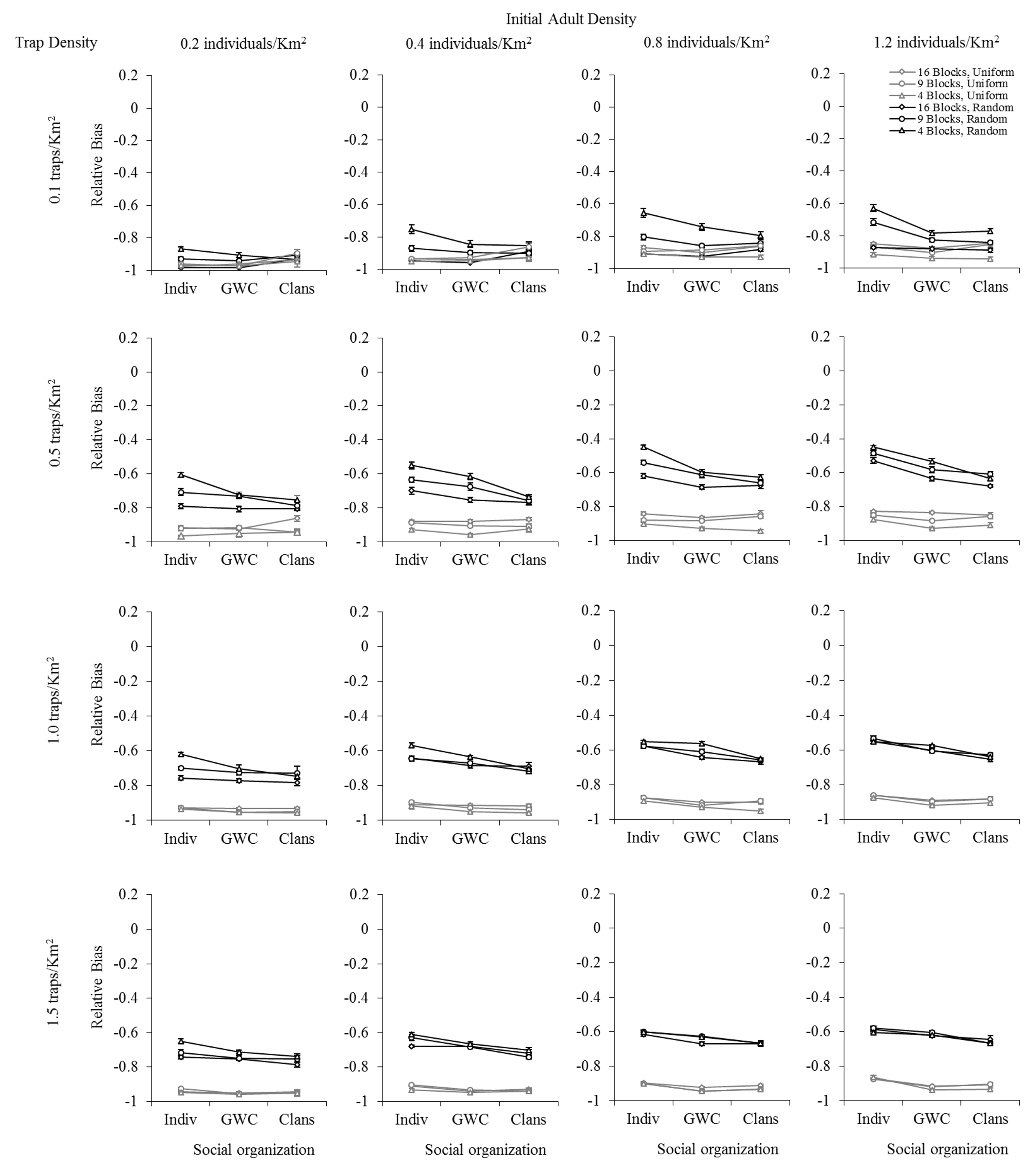
FIGURE B: The effect of trap density, sampling scale, social organization and adult density on mean relative bias in population size estimation using Robust Design. Data shown are for a uniform (grey lines) and random (black lines) trap arrangements, using initial adult densities. Error bars represent standard errors. Social organization: Indiv: non-associating individuals; GWC: groups within clans; Clans: fixed clans.**

**
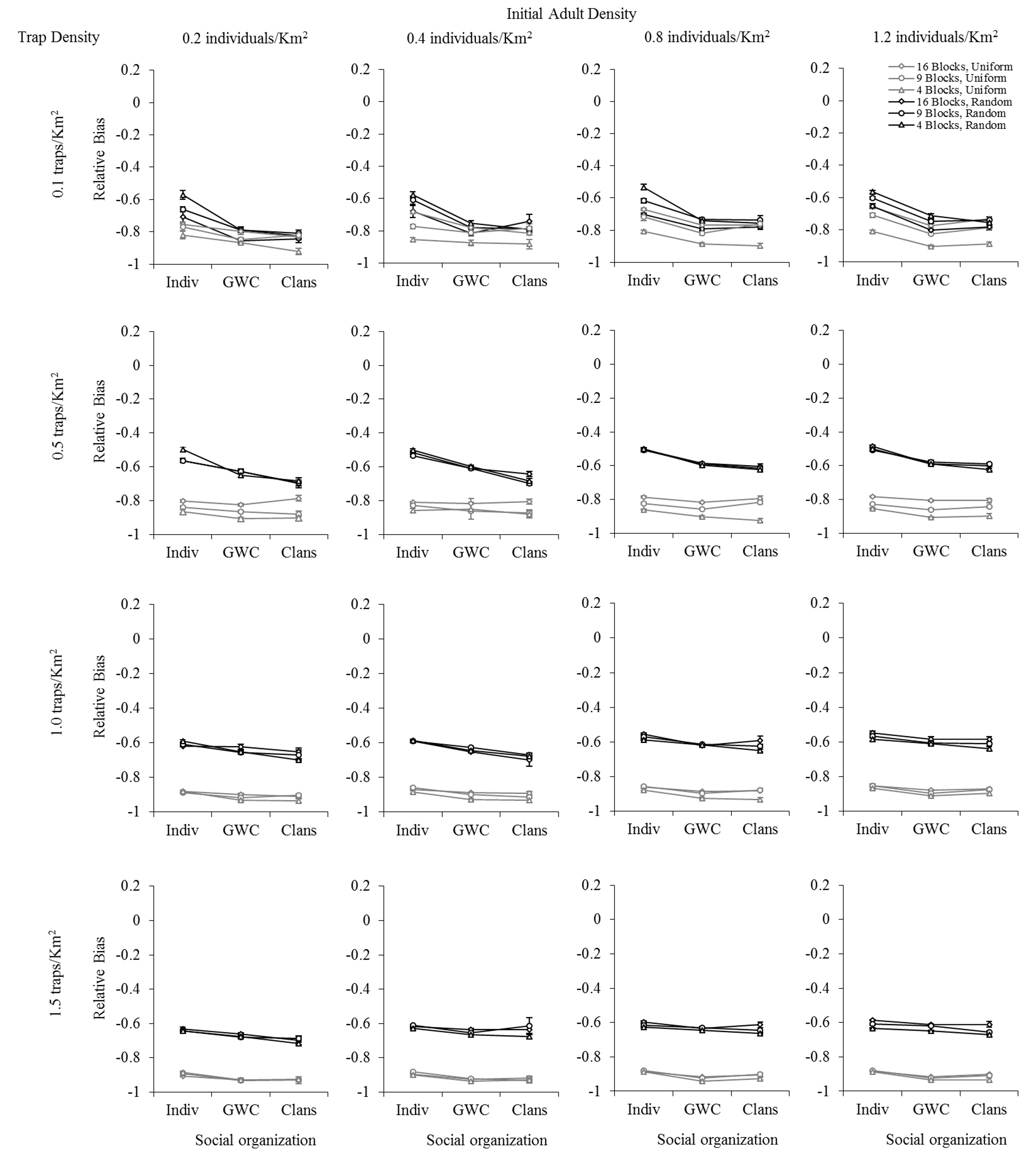
FIGURE C: The effect of trap density, sampling scale, social organization and adult density on mean relative bias in population size estimation using Robust Design with Heterogeneity. Data shown are for a uniform (grey lines) and random (black lines) trap arrangements, using initial adult densities. Error bars represent standard errors. Social organization: Indiv: non-associating individuals; GWC: groups within clans; Clans: fixed clans.**

**
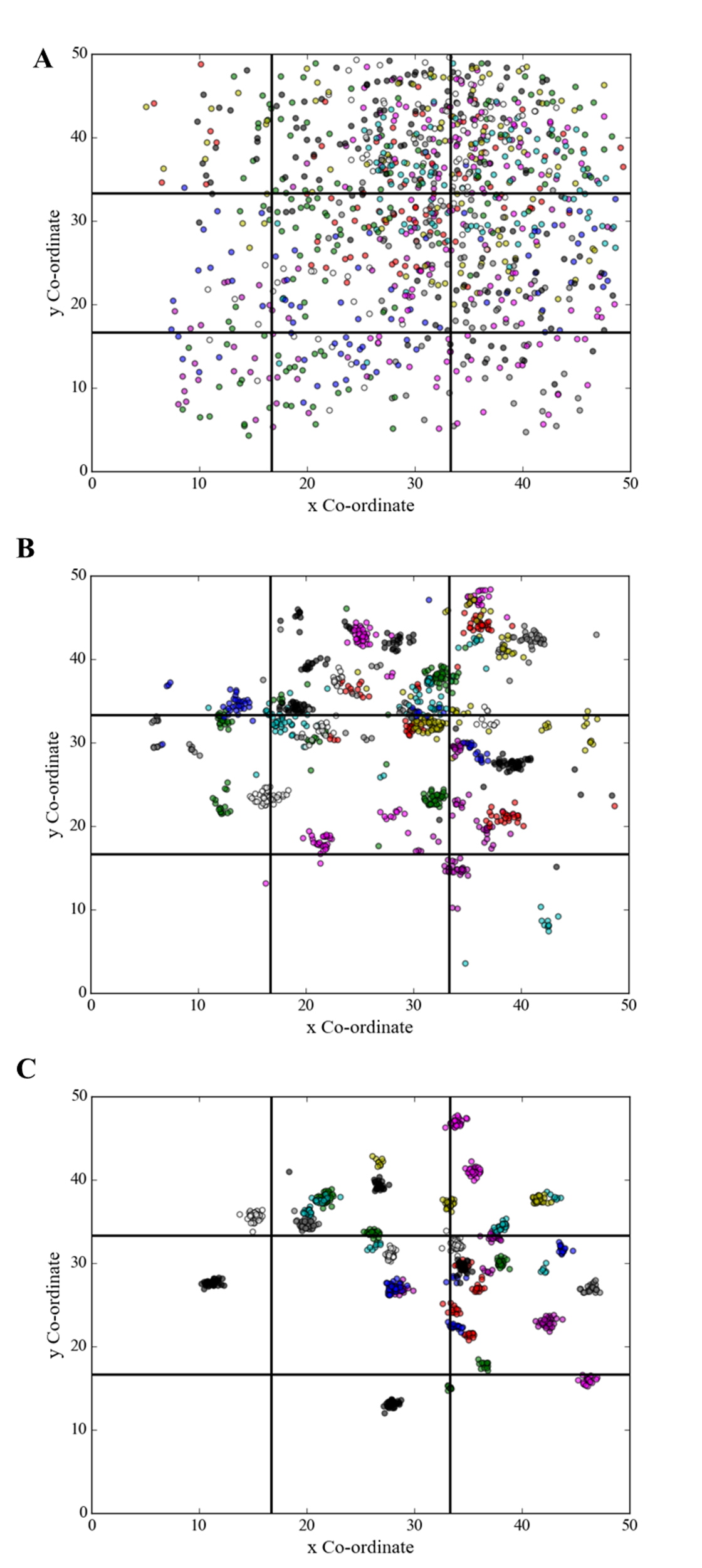
**

**FIGURE D: Snapshots of the positions of individuals during an instantiation of the simulation for the three cases of social organization: A) Non-associating individuals, B) Groups within clans, C) Fixed groups. Each circle in the three figures represents an individual and the colour of the circles indicates clan identity.**
